# A87 PERSPECTIVES ON DIET MODIFICATION TO MANAGE THEIR SYMPTOMS IN PATIENTS WITH INFLAMMATORY BOWEL DISEASE. A SCOPING REVIEW

**DOI:** 10.1093/jcag/gwac036.087

**Published:** 2023-03-07

**Authors:** C V Noejovich, C Yuan, P Miranda, J Szeto, R Patel, D Armstrong, E Verdu

**Affiliations:** Medicine, McMaster University, Hamilton, Canada

## Abstract

**Background:**

The link between diet, disease activity and symptoms in IBD patients have recently gained attention and recommendations on dietary interventions to manage symptoms are common. Most studies have explored the correlation between dietary patterns and increased risk of IBD or symptom severity. However, there is limited understanding and no relevant systematic review of IBD patients’ perspectives and barriers to adopt the prescribed diets. We thus conducted a scoping review for this topic.

**Purpose:**

Aim: We performed a scoping review of current evidence to investigate the extent of evidence on IBD adult patients’ perspectives on dietary modification to manage their symptoms and gaps for future research to explore patients' experiences.

**Method:**

We followed the JBI (Joanna Briggs Institute) method for scoping reviews. A systematic search of Ovid Medline, Embase and Cochrane Library was conducted in April 2022 to retrieve published English language qualitative, quantitative and mixed methods studies that report IBD patients’ perspectives, behaviours, beliefs related to diet modification and barriers to diet adoption for managing their symptoms or disease activity. We manually reviewed reference lists of reviews on this topic. Since this is a scoping review, no statistical comparison is needed.

**Result(s):**

Out of 2822 papers screened, 42 studies met the inclusion criteria. Various methods were used in included studies with heterogenous outcomes reported. Of the 42 studies, 19 reported IBD patients' beliefs and behaviors related to diet as a primary outcome. Most patients reported changing their diet after being diagnosed with IBD, and food avoidance and restrictive diet were commonly reported to prevent relapse. Some studies reported that many patients reduced their opportunities for social life, such as eating out, practicing outdoor sports, having dinner with family in the same household and meeting friends. The dietary modification was more significant among individuals with active than inactive disease. Most studies showed that patients believe food can play a role in causing or preventing relapse, but beliefs are varied regarding the role of diet as initiating factor for IBD. Some patients believe dietary modification could be more important than medication to manage their disease symptoms. Few studies focused on patients' barriers when changing their diet, but financial barriers and limited nutritional guidance were commonly reported acknowledging searching for dietary advice on the internet.

**Conclusion(s):**

Food avoidance and social restriction for relapse prevention are standard practices by most IBD patients. The belief that nutrition is key in managing IBD is prevalent. This scoping review highlights the need to identify patients' barriers to accessing professional dietary guidance and nutritional interventions and provides direction for clinical studies and systematic reviews of focused research questions.

**Please acknowledge all funding agencies by checking the applicable boxes below:**

None

**Disclosure of Interest:**

None Declared

